# Is there a reasonable excuse for not providing post-operative analgesia when using animal models of peripheral neuropathic pain for research purposes?

**DOI:** 10.1371/journal.pone.0188113

**Published:** 2017-11-22

**Authors:** Sara Hestehave, Gordon Munro, Rie Christensen, Tina Brønnum Pedersen, Lars Arvastson, Philip Hougaard, Klas S. P. Abelson

**Affiliations:** 1 Department of Experimental Medicine, Faculty of Health and Medical Sciences, University of Copenhagen, Copenhagen, Denmark; 2 Department of Neurodegeneration In Vivo, H. Lundbeck A/S, Valby, Denmark; 3 Department of Neurology, Danish Headache Center, Glostrup Research Institute, Glostrup, Denmark; 4 Department of Non-Clinical Safety Research, H. Lundbeck A/S, Valby, Denmark; 5 Biometrics, H. Lundbeck A/S, Valby, Denmark; Institut National de la Recherche Agronomique, FRANCE

## Abstract

**Introduction:**

The induction of neuropathic pain-like behaviors in rodents often requires surgical intervention. This engages acute nociceptive signaling events that contribute to pain and stress post-operatively that from a welfare perspective demands peri-operative analgesic treatment. However, a large number of researchers avoid providing such care based largely on anecdotal opinions that it might interfere with model pathophysiology in the longer term.

**Objectives:**

To investigate effects of various peri-operative analgesic regimens encapsulating different mechanisms and duration of action, on the development of post-operative stress/welfare and pain-like behaviors in the Spared Nerve Injury (SNI)-model of neuropathic pain.

**Methods:**

Starting on the day of surgery, male Sprague-Dawley rats were administered either vehicle (s.c.), carprofen (5.0mg/kg, s.c.), buprenorphine (0.1mg/kg s.c. or 1.0mg/kg p.o. in Nutella^®^), lidocaine/bupivacaine mixture (local irrigation) or a combination of all analgesics, with coverage from a single administration, and up to 72 hours. Post-operative stress and recovery were assessed using welfare parameters, bodyweight, food-consumption, and fecal corticosterone, and hindpaw mechanical allodynia was tested for assessing development of neuropathic pain for 28 days.

**Results:**

None of the analgesic regimes compromised the development of mechanical allodynia. Unexpectedly, the combined treatment with 0.1mg/kg s.c. buprenorphine and carprofen for 72 hours and local irrigation with lidocaine/bupivacaine, caused severe adverse effects with peritonitis. This was not observed when the combination included a lower dose of buprenorphine (0.05mg/kg, s.c.), or when buprenorphine was administered alone (0.1mg/kg s.c. or 1.0mg/kg p.o.) for 72 hours. An elevated rate of wound dehiscence was observed especially in the combined treatment groups, underlining the need for balanced analgesia. Repeated buprenorphine injections had positive effects on body weight the first day after surgery, but depressive effects on food intake and body weight later during the first week.

**Conclusion:**

Post-operative analgesia does not appear to affect established neuropathic hypersensitivity outcome in the SNI model.

## Introduction

Neuropathic pain arises from lesion or damage to the somatosensory system and is characterized by an array of positive (eg. allodynia and spontaneous pain) and negative (sensory loss) signs and symptoms [1]. Looking at the patient population living with neuropathic pain, three out of four experience moderate or severe pain despite receiving medication for their pain [2] likely as a consequence of failing to target multiple pain generating mechanisms within individual patients [3]. Accordingly, with this large unmet medical need, a considerable number of nerve injury models in animals have been developed over the past 25 years in an attempt to mimic the clinical manifestation of neuropathic pain. Typically, they have focused on damaging nerves that innervate the hindpaw as this is amenable to showing changes in sensory function via measurement of withdrawal reflexes in response to e.g. mechanical, thermal, or chemical stimulation. These approaches differ in (i) site of injury (distal versus proximal nerve or spinal roots); (ii) nature of injury (ligation, cut, crush or inflammation); (iii) degree of damage (complete vs partial nerve damage), and have thereby yielded individual models with relatively homogeneous animal populations in terms of pathophysiology, albeit this arguably does not translate to the mixed and complex clinical picture of neuropathic pain [4–9].

Irrespective of the method applied, trauma-induced neuropathic models are typically invasive and give rise to peri-operative nociceptive signaling from surrounding tissue, which may be irrelevant to model outcome. Despite this, the provision of appropriate post-operative analgesic regimes are not applied as rigorously as they ought to be [10, 11], despite an irrefutable case from an animal welfare perspective and minimization of experimental variance due to pain-induced stress [12]. Even when the best intentions of the experimenter are applied, the specific analgesic treatment used might not be optimal. Somewhat surprisingly, to date only a handful of studies have investigated this critical issue in animal models of trauma-induced neuropathic pain. Accordingly, whereas post-operative administration of systemically administered opioids or NSAIDs to either Spinal Nerve Ligation (SNL) or Spared Nerve Injury (SNI) rats variously affects the early onset of neuropathic hypersensitivity, at 1–2 weeks post injury these differences are essentially resolved [13, 14]. In contrast, application of a local anesthetic such as lidocaine to the injured sciatic nerve in Chronic Constriction Injury (CCI) rats has been reported to reduce the duration and magnitude of expected hypersensitivity, while it had no influence in the Partial Nerve Ligation (PNL) model [15]. Other publications are even more contradicting, indicating that bupivacaine (which has a more prolonged duration of action than lidocaine) does not affect hypersensitivity outcome parameters in SNI rats in some studies [16], while others do in fact find preventive effects on neuropathic pain behaviors evolving in this model [17]. These observations make it difficult to conclude whether such inconsistencies reflect differences in model pathophysiology or rather deviations in experimental design (e.g. post-operative analgesic used, duration of coverage, dose or route of administration).

The present study used the SNI model and was designed to assess the effects of various analgesic regimens that encompass different pharmacological mechanisms (opioid agonist, NSAID, local anesthetic and combinations thereof) and duration of action, on (i) expected outcome of the model based on hindpaw nociceptive thresholds to mechanical stimulation at different time-points post-injury, (ii) surrogate markers including body weight, food intake, stress hormone levels and welfare scores throughout the 4 week post-injury period. We found that none of the chosen regimens inhibited the development and maintenance of mechanical allodynia in SNI rats *per se*. However, specific multimodal regimens expected to deliver optimal post-operative analgesia impacted negatively on some welfare aspects of the animals. Although presumed optimization of surgical analgesia does not necessarily equate directly into welfare benefits, our findings clearly indicate that this should not preclude a minimal provision when working with animal models of neuropathic pain given that behavioural outcome is unaffected.

## Materials and methods

The following study was performed in accordance to the Danish legislation (Law no. 474 of May 15th, 2014 and Order no. 12 of 07/01/2016) regulating experiments on animals, which is in compliance with the European Directive 2010/63/EU. Experimental protocols for the SNI procedure and following testing at H. Lundbeck A/S were approved by the Danish Animal Experiments Inspectorate.

### Animals and housing

Male Sprague-Dawley rats (Crl:CD (SD)) from Charles River, Germany, weighing 226 ±18g (mean ± standard error of the mean [SEM]) on the day of surgery were used. Animals were housed in pairs (with the exception of a few animals who lost their partner during anesthesia, Table 1), in transparent polycarbonate macrolone III-high open cages (Scanbur, Denmark, 42.5 * 26.6 * 18.5 cm) with enrichment consisting of aspen wood chewing blocks (S-Bricks from Tapvei, Estonia), paper-wool shavings (LBS Biotechnology, UK) for nesting material and red Rat Retreats^TM^ (Bio-Serv, Flemington, US) for hiding. For bedding, aspen chips (Tapvei, Estonia) were used. Cages and bedding were changed daily when collecting fecal samples and cleaned twice a week in periods where feces was not collected daily. Food (Altromin 1324, Brogaarden, Denmark) and water were available ad libitum, and water was changed on a weekly basis. The light-dark cycle was 12:12h with lights on from 6.00; the room temperature was set to 20 ± 2°C; and the relative humidity was 30–70%.

**Table 1:** Treatment- and non-operated control-groups.

Group	Analgesic/dose	Administration	Treatment timing ^1^	Abbreviation	Coverage / hours	N	N, end
A	Control (minimally handled and not operated)	-	-	Control	-	18	18
B	Control + von Frey tested (not operated)	-	-	Control + VF	-	14	14
C	Vehicle (saline)	s.c.	During anesthesia, prior to incision	Vehicle, s.c.	-	30	30
D	Buprenorphine, 0.1 mg/kg	s.c.	During anesthesia, prior to incision	Bup. s.c. 8h	8	30	30
E	Buprenorphine, 0.1 mg/kg	s.c.	During anesthesia, prior to incision + every 8 h	Bup. s.c. 72h	72	12	12
F	Buprenorphine, 1.0 mg/kg	Nutella/p.o.	45min prior to anesthesia	Bup. p.o. 8h	8	12	12
G	Buprenorphine, 1.0 mg/kg	Nutella/p.o.	45min prior to anesthesia + every 8 h	Bup. p.o. 72h	72	14	13*
H	Carprofen, 5.0 mg/kg	s.c.	During anesthesia, prior to incision	Carprofen, 24h	24	12	12
I	Carprofen, 5.0 mg/kg	s.c.	During anesthesia, prior to incision + every 24h	Carprofen, 72h	72	12	11**
J	Lidocaine 10 mg/Bupivacaine 2.5 mg, 1:1	Local	During anesthesia, post incision.	Lido/bupi	2–3	12	12
K	**High dose combination:** Carprofen, 5 mg/kg. Buprenorphine 0.1 mg/kg. Lidocaine10 mg/ bupivacaine2.5 mg– 1:1	s.c./local	During anesthesia, prior to incision + local post incision.	High combination	72	18	12*,**
L	**Low dose combination:** Carprofen, 5mg/kg. Buprenorphine 0.05 mg/kg. Lidocaine10 mg/ bupivacaine2.5 mg– 1:1	s.c./Local	During anesthesia, prior to incision + local post incision.	Low combination	72	12	12

^1^All analgesics supplied subcutaneously (s.c.) were given when the animal was anesthetized, prior to shaving and surgery. Buprenorphine administered p.o. in Nutella was administered approximately 45 min prior to surgery. Local irrigation of lidocaine/bupivacaine in the incision site was made after localization of the nerves but prior to ligation and axotomy.

* Rat died during anesthesia (incidental loss).

**Rat(s) euthanized due to complications / side-effects to the analgesic protocol. Accordingly, two rats originally placed in Group B, were moved to groups K & I respectively due to anesthetic deaths.

### Groups and study design

A total of 228 rats representing five sequential cohorts were tested over a period of 4 months following allocation using a block design into twelve groups consisting of 10 treatment and 2 non-operated control groups (Table 1). All testing, scoring and administration of analgesics during this study was performed by the same female investigator with no pre-determined blinding of treatment groups performed. Rats were acclimatized to the conditions in the animal facility for at least one week after arrival from the vendor, following which they were moved to the testing laboratory within the same facility at least three days prior to baseline testing. From thereon, they stayed in the same room during the entire study and were only moved to another room temporarily to perform the SNI surgery. Baseline measurements for mechanical allodynia were obtained two days prior to the surgical procedure and minor rearrangements in the groups were made in order to align baseline means in response to mechanical stimulation. Animals receiving their treatment per orally (p.o.) in Nutella^®^ (Ferrero Scandinavia AB, Sweden), were habituated to ingestion of this twice a day for three days prior to SNI surgery. Mechanical thresholds, body weight, food and water intake, and welfare scores were measured on Day 0 (except mechanical threshold, which was not measured on the day of surgery), 1, 2, 3, 5, 7, 14, 21 and 28 post surgery. Fecal-samples covering 24 hours of defecation for measurements of immunoreactive fecal corticosterone metabolites (FCM) were collected at the same time in the morning of Day 0, 1, 2, 3, 5, 7 and 28 post surgery. At the end of each experiment, all animals were euthanized with 80% CO_2_ and 20% O_2_ for the first 30 seconds, with a gradual increase to 100% CO_2_ for 7 minutes. Euthanasia was verified by a lack of blood circulation. Necropsy was performed on at least half of the animals in each group. In groups where animals at some stage in the trial had clinical symptoms or signs of discomfort, necropsy was performed on all animals.

### Surgery

The surgical procedure was based on the method described by Decosterd & Woolf, 2000 [8], and the same experienced technician performed all surgeries. Anesthesia was induced with 5.0–5.5% sevoflurane delivered in a mixture of 70% O_2_ and 30% N_2_O in a Plexiglas induction chamber and maintained by 2.0–3.0% sevoflurane in a 70:30-mixture of O_2_/N_2_O supplied via a face mask for spontaneous breathing. The quality of anesthesia was monitored regularly by observing respiration, oxygenation by means of skin-color, and testing the hind paw withdrawal reflex. The rat was placed in left lateral recumbence, the fur was shaved on the lateral surface of the right thigh and the area was swabbed with iodine to secure aseptic conditions. A longitudinal incision was made through the skin caudal to the femur, and the underlying musculature (*musculus biceps femoris*) was opened with scissors and blunt dissection to reveal the sciatic nerve and the three terminal branches; the sural, common peroneal and tibial nerves. A gentle pinch with forceps was performed on the common peroneal and tibial nerves before ligation to verify that they were the intended nerves; pressure of the common peroneal nerve should provoke a brief flick of the toes, whereas the tight ligation of the tibial nerve provokes twitches in the surrounding musculature [18]. The common peroneal and the tibial nerves were ligated with 5–0 Prolene ligatures (Jørgen Kruuse A/S, Denmark), and sectioned distally to the ligation, removing approximately 2 mm of the distal nerve stump, thereby performing both axotomy and ligation of the two nerves, and leaving the sural nerve intact. Great care was taken not to irritate, stretch or pull the intact sural nerve. The musculature was reattached and the skin closed with tissue glue (Vetbond^TM^, Jørgen Kruuse A/S, Denmark) or simple interrupted sutures (4–0 vicryl, Jørgen Kruuse A/S, Denmark) in animals receiving lidocaine/bupivacaine irrigation. At the end of surgery, all animals were treated with 10 mg/kg antibiotics (Baytril®, Bayer AG, Germany) subcutaneously (s.c.) to reduce the risk of post-operative infections, and analgesics or vehicle according to their specific treatment-group as described in Table 1. Notably, rats receiving buprenorphine p.o. in Nutella^®^ were treated approximately 45 minutes prior to surgery, whilst lidocaine/bupivacaine irrigation was applied at the incision-site and covered the nerves for 2–3 minutes before ligation and axotomy, and left in the incision-site upon closure. The duration of the procedure, from induction of anesthesia until the animal regained consciousness was approximately 15–20 minutes. Animals were examined routinely for signs of distress, wound dehiscence or wound infection post-operatively.

### Measurement of mechanical allodynia

Behavioral assessments and testing of the different groups of animals were performed between 08.00–14.00 h by the same female experimenter and in the same order every test day. Mechanical allodynia was assessed by using a series of calibrated von Frey monofilaments (North Coast Medical, Inc., Morgan Hill). Animals were placed in individual Plexiglas (10.8*13.8*17.0cm) enclosures on an elevated wire grid located in the same room in which they were routinely housed. They were given at least 15 minutes to acclimate to the enclosure and the experimenter’s presence prior to stimulation of the lateral plantar surface of the hindpaw, at different anatomical locations in the area innervated by the intact sural nerve, with a series of calibrated von Frey filaments (0.4, 0.6, 1.0, 1.4, 2.0, 4.0, 6.0, 8.0, 10.0, 15.0, 26.0 g). To initiate testing a filament with a bending force of 4.0 g was first applied to the hindpaw with uniform pressure for 5 seconds. A brisk withdrawal to at least three out of five applications was considered a positive response whereupon the next lower filament in the series was applied. In the absence of a positive response the neighboring higher filament was applied. Using this paradigm progression through the sequence of filaments in either ascending or descending order was continued until the threshold for induction of a positive reaction was reached. The threshold was set as the lowest force that evoked a brisk withdrawal response to three of five consecutive applications. Lifting of the paw due to normal locomotor activity was ignored. Care was made to ensure that rats were not distracted or sleeping while being stimulated by the von Frey filaments.

### Welfare score

Each rat was given a welfare score on each test day, as well as on the day of the baseline test prior to surgery using the animal welfare score sheet presented in Table 2 [19].

**Table 2:** Animal welfare score sheet.

Parameter	Score	Observation
General	0.0	Awake, active, responding
Condition	0.1	Buries in bedding, hiding, lying still but runs off when touched
	0.4	Sitting still, unwilling to move, buries/hides. Presses head towards cage floor, frightened and /or unusually aggressive when being touched
Porphyria	0.0	No coloured secretion, clear and clean eyes
	0.1	Mild porphyria, gucky eyes and nose
	0.4	Pronounced porphyria in face or on paws
Movement	0.0	Normal
and body posture	0.1	Minor incoordination when animal is being stimulated; crouched; mild piloerection
	0.4	Pronounced ataxia, head kept crooked; crouched; one or both legs dragged behind, pronounced piloerection
Wounds	0.4	Bites or scratches itself, leading to wounds
	0.4	surgical wound not healing; sutures bursting
Body	0.0	<5% weight loss compared to pre-procedure
Weight	0.1	5–10% weight loss compared to pre-procedure
	0.4	10–20% weight loss compared to pre-procedure
Appetite	0.0	Normal food intake; normal urination and defecation
	0.1	No sign of food intake; consumes water, no signs of dehydration
	0.4	No sign of food intake or water consumption, signs of dehydration.
Total score;		

Originally developed by Hampshire and co-workers (2001)[19].

The animals were evaluated for 15 min when removed from their home cages during the habituation period prior to von Frey testing (six animals per session). If the score exceeded 0.3 and was unrelated to wound dehiscence, the veterinarian of the animal facilities was consulted. In the case of wound dehiscence, the animal was re-anesthetized and re-sutured, with Fuciderm®Vet-crème (Dechra Veterinary Products, England) applied to the incision site.

### Measurement of immunoreactive fecal corticosterone metabolites (FCM)

On the days selected for measurement of FCM, all bedding was changed in the morning, covering a 24-hour period, and kept at -20°C until sorting of fecal pellets from the bedding material. After sorting the pellets from the bedding material, pellets were immediately stored at -20°C until analysis. FCM were analyzed according to a previously described method [20, 21], with some modifications regarding evaporation. In brief, all fecal boli from the specific cage and day were weighed and submerged in 96% ethanol (5 ml/g feces). Each sample was incubated at room temperature on a shaking table overnight. The samples were processed in a BagMixer (BagMixer^®^ 400CC, Interscience, Saint Nom, France) in 2x210 seconds to homogenize the ethanol-feces-solution. The solution was filtered free of fecal material and approximately 13 ml of the filtered solution was moved to a tube and centrifuged in a Scanspeed 1236p centrifuge (LaboGene Aps, Lynge, Denmark) for 20 minutes at 2.000 rpm. The supernatant was decanted and re-centrifuged for another 20 minutes as described above. A 1.5 ml aliquot of the supernatant was transferred to an Eppendorf tube and further centrifuged at 10.000 rpm for 15 minutes in a tabletop Eppendorf centrifuge 5415D (Eppendorf AG, Hamburg, Germany). One ml of the supernatant was stored at -20°C until further analysis. For analysis, 300 μl of the supernatant was processed in an evaporator (Genevac EZ-2 personal evaporator, Stone Ridge, NY, USA) for approximately 2 hours. The evaporation was followed by adding 300 μl PBS to each sample along with three to four 2 mm solid-glass beads (Sigma-Aldrich, St Louis, MO, USA) and placed on a shaking table for 2 hours prior to FCM quantification. The samples were quantified using the DRG-Diagnostics Corticosterone ELISA (EIA-4164; DRG Instruments GmBH, Maburg, Germany) according to the manufacturer’s instructions. The absorbencies were recorded at 450nm (reference wavelength, 650 nm), using a Thermo Multiscan Ex micro plate reader (Thermo Fischer Scientific Inc., Waltham, MA, USA).

### Drugs and administration protocols

Doses used for drug treatments in the current study were based on published recommendations unless stated otherwise [22] with supplier details provided in Table 3. A local-anesthetic mixture was made using 10 mg/ml lidocaine (1%) + 2.5 mg/ml bupivacaine (0.25%) in a 1:1 ratio [23], with approximately 0.1 ml of the solution used to irrigate the incision site at the time of surgery. The partial mu-opioid receptor agonist buprenorphine was sourced separately for s.c. and p.o. administration from RB Pharmaceuticals Limited (U.K.; Temgesic®) and from Sigma-Aldrich respectively. Typically, for s.c. administration of buprenorphine a dose range of 0.02–0.05 mg/kg is used [22]. However, other studies have suggested that higher doses may be necessary for optimal analgesic coverage [24]. We have recently assessed the analgesic efficacy of buprenorphine (0.015–0.3 mg/kg, s.c.) administered subcutaneously in naïve rats using the hot plate test [25] and based on these studies, we selected a 0.1 mg/kg dose to administer to SNI rats in order to get robust analgesic coverage [25]. For p.o. administration in Nutella^®^ (Ferrero, Nestle), rats were habituated to voluntary ingestion of 2 g Nutella^®^/kg BW [26] by placing the nut paste on a piece of adhesive tape in their homecage environment twice a day for three days prior to surgery, similarly to the methods presented in Hestehave et al. 2017 [25]. For the first day pair-housed rats were habituated together, and then for the next two days prior to surgery, one rat was moved to another cage in order to ingest the required amount of Nutella^®^. For each habituation session, some bedding material and the red Rat retreat^®^ were moved together with the rat, to help minimize the stress associated with different environmental cues. Thereafter, each rat was administered its individual amount of Nutella^®^, while separated, and was then reunited with its original homecage partner. On the last day as well as the day of the surgery, upon separation, all animals ingested their entire individually offered dose of Nutella^®^ within 3–5 minutes of separation before being returned to their homecage. Standard pharmacokinetic indexes suggest that oral doses of opioids should be in the order of 10 times the parental doses to compensate for bioavailability differences following first-pass metabolism [27]. Antinociceptive effects in the hot-plate test have previously confirmed that 1.0 mg/kg buprenorphine in Nutella^®^ is equi-efficaceous with 0.1 mg/kg s.c. administration, and was therefore used in this study [25]. The preparation for voluntary ingestion of buprenorphine mixed in Nutella^®^ was carried out according to the recommendations made by Abelson et al. 2012 [26] and the habituation- and administration-procedure similar to Hestehave et al. 2017 [25]. With a reported duration of analgesic action of 8–12 hours [10], buprenorphine was re-administered every 8 hours for up to 72 hours to extend coverage through the post-operative period. Similarly, the NSAID carprofen (Rimadyl vet. 50 mg/ml) was re-administered every 24 hours for up to 72 hours. All analgesics administered by a subcutaneous route of injection were diluted with saline so that the concentration corresponded with an administration of 5.0 ml/kg body weight solution. Analgesics, antibiotics and manufacturer details are presented in Table 3.

**Table 3:** Drugs and manufacturer.

	Product	Manufacturer
Vehicle	Nutella^®^	Ferrero, Nestle
	Saline = sodium chloride 9 mg/ml	Fresenius Kabi.
Opioid	Buprenorphine-Hydrochloride-powder ^1^	Sigma-Aldrich, Inc.
	Buprenorphine/Temgesic^®^ ^2^	RB Pharmaceuticals Limited, England.
NSAID	Carprofen/Rimadyl vet.^®^ 50 mg/ml	Pfizer Inc., NY, USA.
Local analgesic	Lidocaine/Xylocaine^®^ 10 mg/ml	AstraZeneca
	Bupivacaine/Marcain^®^ 2.5 mg/ml	AstraZeneca
Antibiotic	Enrofloxacin/Baytril vet. 50 mg/ml	Bayer A/S.

^1^: Powder-formulation used for Nutella^®^-mixture, and also for s.c. in saline administration in cohorts including Nutella^®^ administration. This was done in order to secure administration of the exact same active compound

^2^: Used for s.c. in saline administration in cohorts not including administration of buprenorphine in Nutella^®^.

### Statistical analysis

Before initiation of the study, animals were randomly allocated to treatment in pairs of two, so that cage-mates were allocated to the same treatment-group, as measures of FCM and food intake were performed on cage-level, and not the individual animals. Minor rearrangements in group allocation were made according to individual von Frey threshold values obtained prior to surgery in order to ensure equivalent baseline group means within each cohort. Statistical analysis was performed using GraphPad Prism version 5 (GraphPad Software, Inc., La Jolla, CA, USA), and SAS version 9.4 (SAS Institute Inc., NC, USA). Statistical comparisons on overall treatment effects between groups were made using Repeated Measures Two Way ANOVA for food intake (using GraphPad). Changes in von Frey threshold between baseline and Day 28 was analyzed using Mixed Model and Tukey’s Test on delta-values (Day 28 minus baseline) (using SAS). Analysis of body weight was done by a repeated measurements model with fixed effects of treatment (factor), time (factor) and baseline body weight. Also an interaction between treatment and time was included as well as an interaction between baseline body weight and time. After a request by a reviewer to also account for a potential effect of cage-mates, a more complex model was set up using a covariance structure based on the Kronecker product of a factor specific covariance matrix modelling the effect of time combined with a correlation model between the two animals in the cage [28], using SAS proc mixed. Statistical comparison between groups were made using Bonferroni post-test and comparison against the non-operated control group A (88 tests obtained from 11 treatment comparisons across 8 time points). A similar calculation was done comparing to the vehicle-treated group C. In the Mixed Model for von Frey data a random effect was also introduced to take into account the influence it might have that rats are grouped in pairs as cage-mates receiving the same treatment, but as the results obtained for food-intake were based on cage-level recordings, this was not necessary in the analysis of these results. P<0.05 was considered statistically significant.

## Results

### Mechanical allodynia

**Fig 1:**
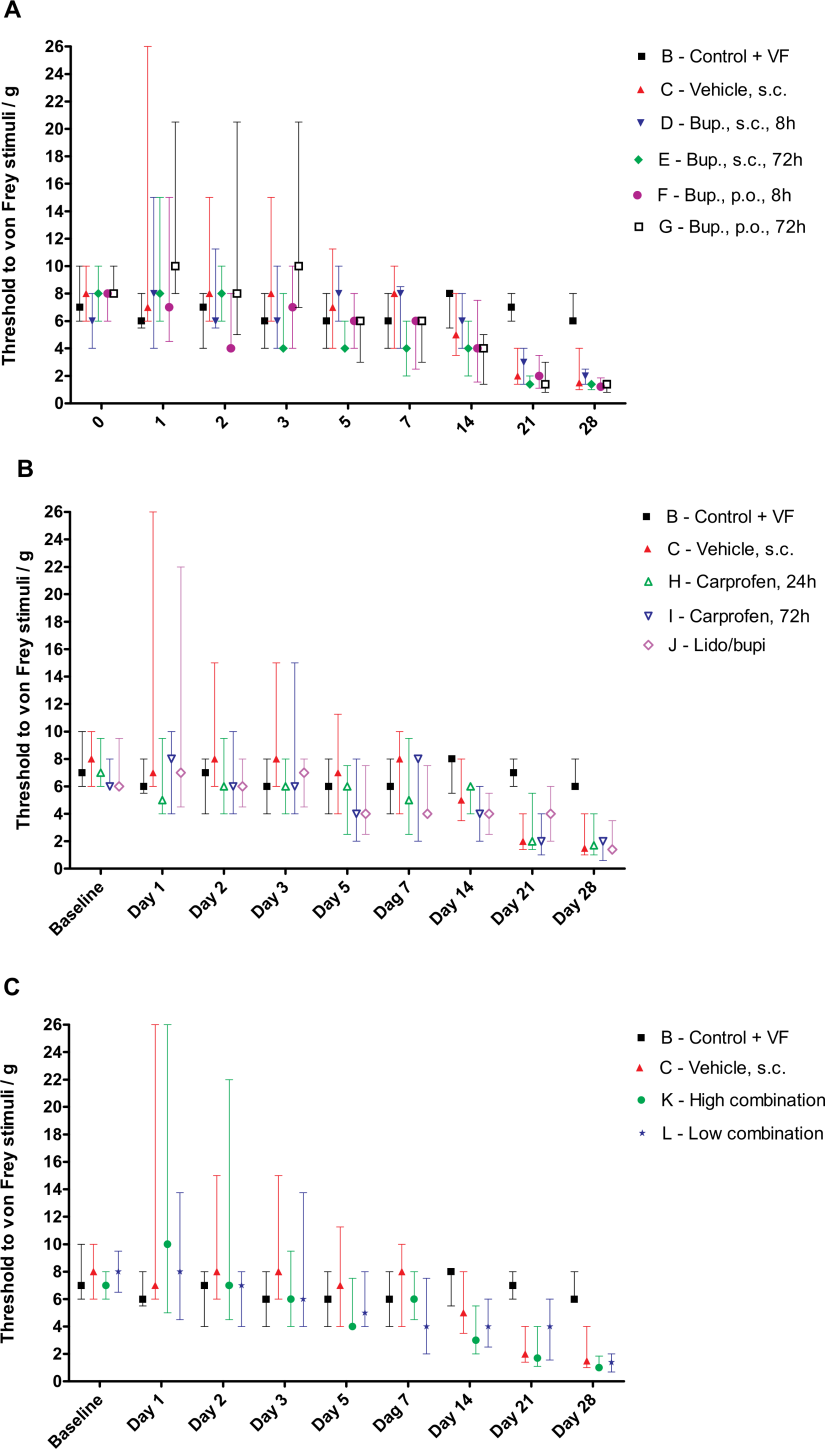
Development of mechanical allodynia in SNI rats. Panels A-C Measurements of mechanical allodynia by von Frey monofilaments on day 1, 2, 3, 5, 7, 14, 21, 28 post SNI procedure. Presented as median ± interquartile range of von Frey measurements. Groups and treatment doses are presented in Table 1**. (A)** Presenting treatment groups receiving buprenorphine alone or vehicle and the non-operated control group. (**B)** Presenting treatment groups receiving carprofen, lidocaine/bupivacaine or vehicle and the non-operated control group. (**C)** Presenting treatment groups receiving combined treatment or vehicle and the non-operated control.

All treatment groups displayed an overall decrease in withdrawal threshold to von Frey stimulation of the injured hindpaw as shown in Fig 1. Although only 2/184 operated rats (one rat each in Groups H and J respectively) failed to show any signs of mechanical allodynia throughout the test-period compared to their pre-surgery baseline, Fig 1 clearly indicates that there was a large inter-individual variation within groups when tested from Days 1–7 post injury despite relatively similar median values.

Statistical comparison was performed on delta-values (Day 28 minus Baseline) using a Mixed Model with Tukey’s Test to compare groups. In the mixed model, a random effect, to compensate for possible effects that cage-mates could have on one another [29] was introduced (performed in SAS). No statistically significant differences were found between any of the operated groups on day 28, indicating that none of the post-operative treatments tested affected model outcome. No positive correlation was found between cage-mates, indicating that allocation of cage-mates to the same treatment, did not modify our results. These results were confirmed when the same statistical analysis was performed on data from Day 28 instead of an analysis based on delta-values; all groups were significantly different from the non-operated control group (B), and no statistically significant differences were detected between any of the operated groups.

### Body weight

**Fig 2:**
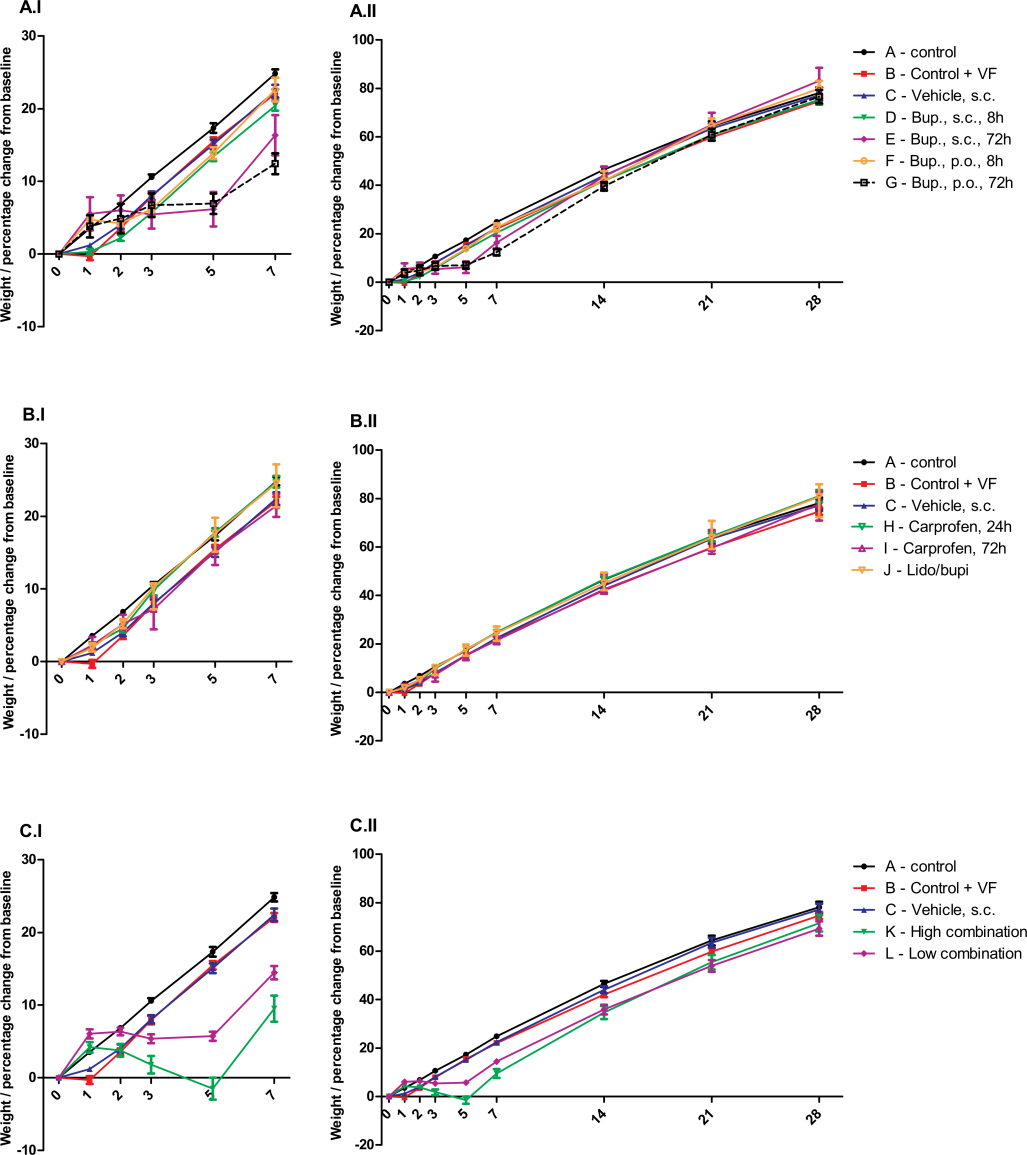
Change in body weight following SNI-surgery. Figure shows the percentage weight change in SNI rats compared to Day 0, from Day 0–7 in version I, where the greatest differences occur, and Day 0–28 in II. Data is presented as mean ± S.E.M. Groups and dosing details are presented in Table 1. For panels A-C statistical comparisons were made for each treatment group versus the vehicle treated group (C) using a repeated mixed model with a correlation model for “cage-mates” and Bonferroni post-tests; *P<0.05, **P<0.01, ***P<0.001. Statistical differences are not presented in the figure. Significantly lower body weight than vehicle treated group (C); (**A.I+II)** Control, vehicle and buprenorphine-groups. Group E, Bup., s.c., 72 hours; Day 5; ***, Day 7; **. Group G, Bup., p.o., 72 hours; Day 5; ***, Day 7; ***. (**B.I+II)** Control, vehicle, carprofen and lido/bupivacaine-groups. No statistical significant differences were recorded. (**C.I+II)** Control, vehicle and combined treatment groups. K–High Combination; Day 3; ***, Day 5; ***, Day 7; ***, Day 14; ***. L–Low combination; Day 5; ***, Day 7; ***. Following groups was statistically higher than the vehicle-treated group on day 1; Group E (*), K (*) and L (***).

Since the animals starting out with slightly different baseline-weights, were young and transitioning through a period of rapid growth, the influence of surgery/treatment on body weight is illustrated as percent change compared to weight on the day of surgery (Fig 2).

Body weight was analyzed using a repeated measures mixed effect model, combining dependence over time with a correlation for cohoused animals. This correlation was found to be small (0.111 ± 0.041). Statistical comparison between groups were made using Bonferroni post-test and comparison against both the non-operated control group A, and the vehicle-treated group C. The largest differences between treatment groups were observed in the first week post-surgery as shown in Fig 2, A-C.I. This clearly shows that groups receiving buprenorphine for 72 hours, both when administered in combined treatment groups or alone in Nutella^®^ or saline, all stopped gaining or even started to lose weight after Day 2. Particularly, the group receiving the high combination (K) demonstrated a significant decrease in body weight from Day 3. On Day 5, this group had actually lost 1.5±1.5% (mean ± S.E.M) compared to Day 0, whereas the control group (A) had gained 17.3 ± 0.7%. After this, the high combination group started to gain weight, but remained significantly lower than control until day 21. The rats receiving the low combination of drugs (L), with the lower dose of buprenorphine (0.05 mg/kg, s.c.), also showed a statistically significant difference from the control group (A) from Day 3 to 21, though with a weight gain of 5.7±0.6% (mean ± S.E.M) on Day 5 and only increasing from this point. The two buprenorphine 72 hour groups (E and G) were both significantly lower than the control-group (A) on Day 5 and 7, and Group E also on day 3, but all groups caught up with the other groups in the last weeks of the experiment. Similar changes were also found when comparing the operated groups to the operated vehicle group (C). All of the groups receiving buprenorphine for 72 hours either alone or in combination with other analgesics (E, G, K and L) demonstrated statistically significant lower levels of body weight gain than vehicle treated animals; the high combination group (K) displayed significantly lower weight gain levels on Day 3–14 while the low combination group (L) and the pure buprenorphine groups (E and G) were only significantly lower on Day 5 and 7 post-surgery. Of special interest, three of these groups (E, K and L) actually exhibited improved weight gain effects on Day 1 compared with vehicle-treatment. All the remaining groups receiving either vehicle, a single administration of buprenorphine (D and F), carprofen (H and I) or local analgesics gained weight similarly to the minimally handled control group (A).

### Food consumption

**Fig 3:**
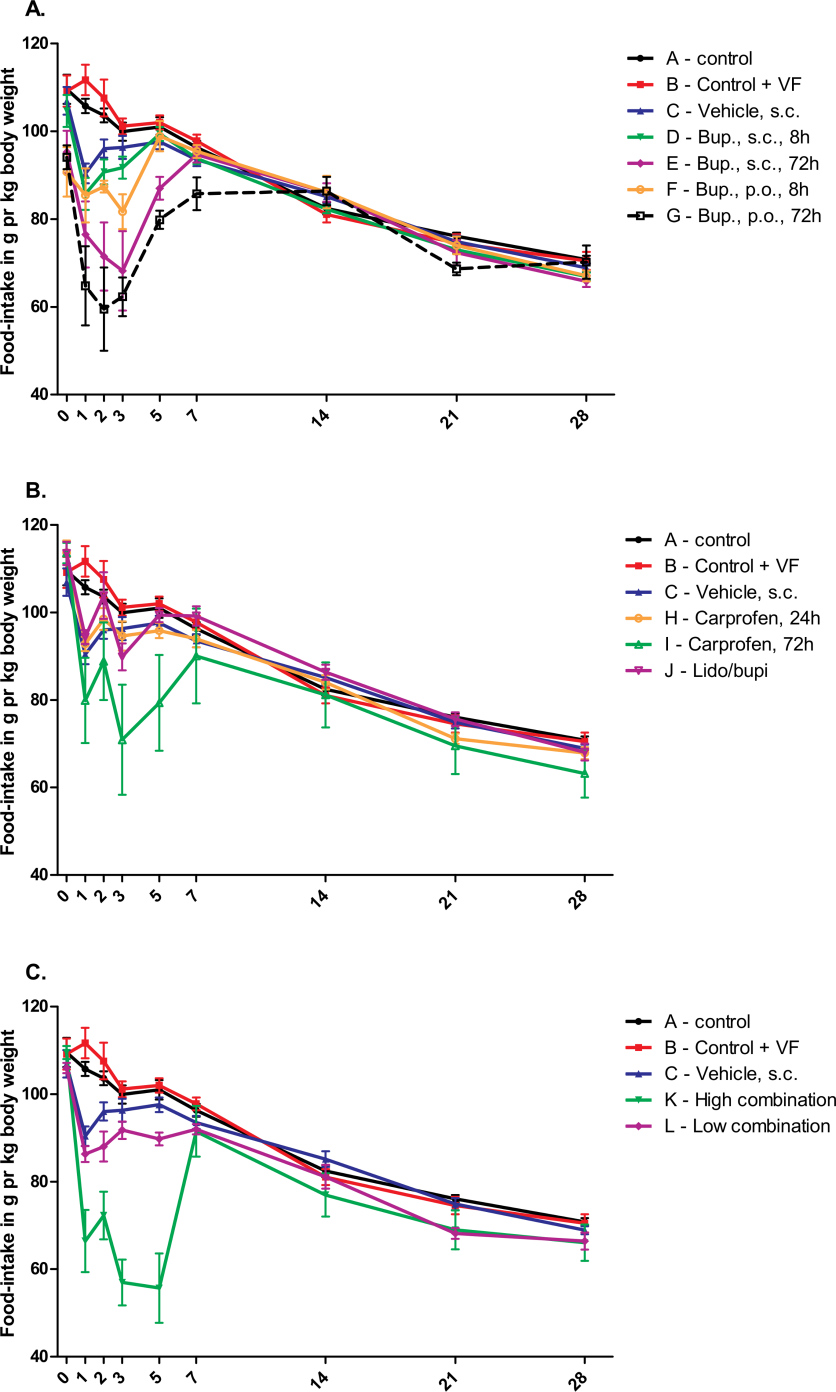
Effect of SNI procedure on food consumption compared to animal weight. The figure shows the amount of food-consumption per kg of animal (combined body weight of co-housed rat pairs) per 24 hours on Day 0, 1, 2, 3, 5, 7, 14, 21 and 28, and is presented as mean ± S.E.M. Groups and treatment doses are presented in Table 1. Multiple comparison was performed by two way Repeated Measures ANOVA and Bonferroni post-tests, and results are presented in the text.

Overall food consumption is presented as an average intake during 24 hours per kilogram of animal in the different treatment groups in Fig 3. As animals were pair-housed with partners given the same treatment, the data presented is the combined food intake in the cage per weight of the two rats habituating the cage. Already on Day 0 (before surgery), there was a significant difference between some of the groups (F and G) compared to the minimally handled control group (A). It was expected that groups F and G would be lower than controls, as they had been fed/habituated to the high-energy nut-paste, Nutella^®^ the previous days, and therefore may have had some of their energy requirements from this source. On Day 1 post surgery, almost all operated groups were significantly different from the control group (A), as expected following anesthesia and surgery, with the exception of the groups receiving 24h carprofen (H) and local analgesia (J). On Day 2 all groups treated with buprenorphine (D, E, F, G, K and L) were still significantly lower than control (A), whereas the remaining groups were similar to the control group. As with the body weight changes, the three groups receiving the high dose of buprenorphine for 72 hours, either in saline or Nutella^®^, alone or in combination (E, G and K), decreased significantly in food consumption over the first three (E) to five (G and K) days, while the group receiving only a single administration of buprenorphine in Nutella (F) decreased for the first three days (Fig 3). On Day 3 and 5, the carprofen 72 hours treatment group (I) was significantly lower than control. The only two groups that did not display a decrease in food consumption after surgery, were the carprofen 24 hour treated group (H) and the group receiving local analgesics (J). When comparing the treatment groups with the vehicle group (C) no group showed a significantly higher food consumption.

### Welfare scores

**Fig 4:**
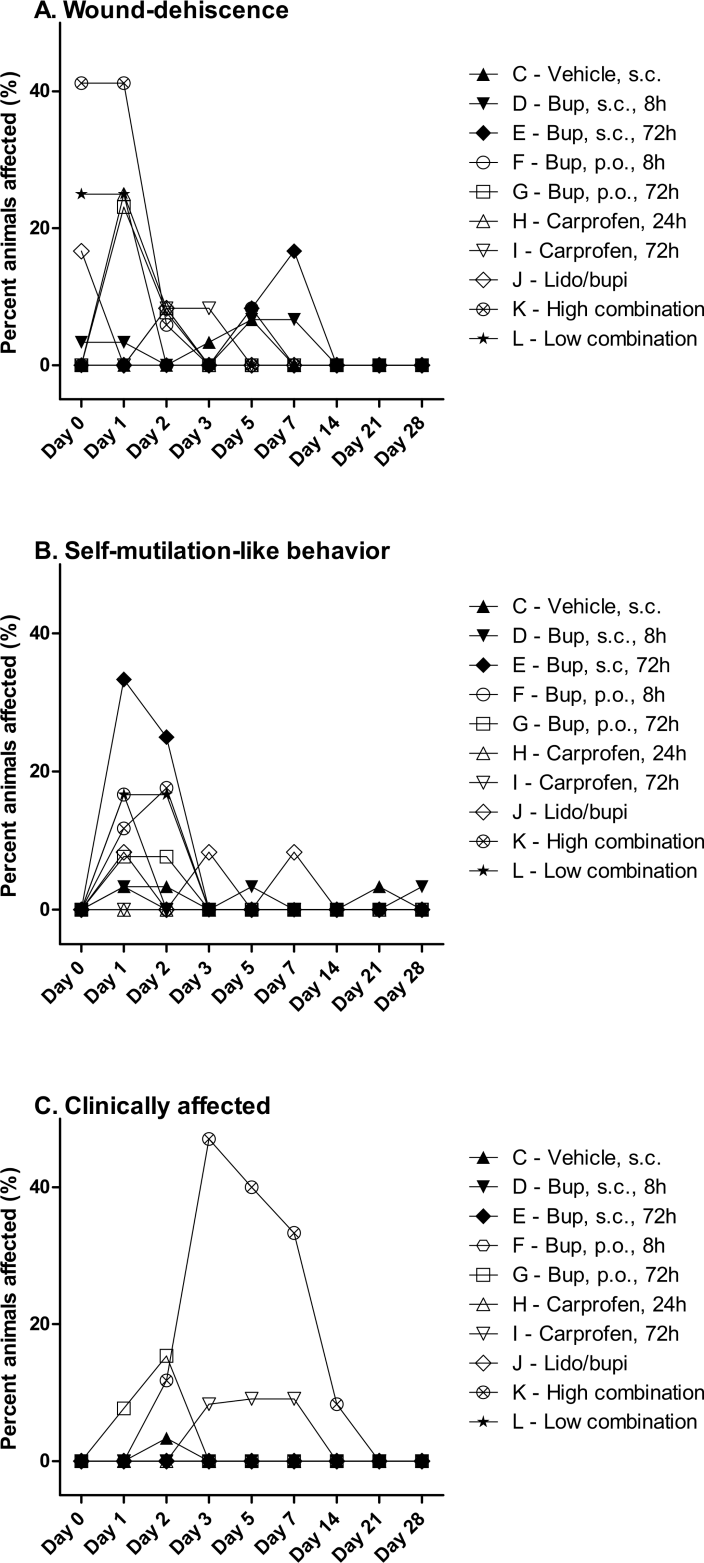
Quantitative comparison of welfare issues experienced by SNI rats allocated different analgesic regimens as summarized in Table 4. Panels **A-C** show the percentage of animals in a given treatment group from Days 0–28 post surgery experiencing (**A)** wound dehiscence, (**B)** self-mutilation-like behaviors characterized by minor wounds, erosions or swelling of the affected paw, (**C)** that were clinically affected as characterized by signs or symptoms such as depressive-like behaviour, decreased food intake, >5% weight loss, piloerection, crouched stance, ataxia, or generalized inactivity.

Throughout all the treatment groups wound dehiscence was observed in several animals. This was especially prevalent in the group receiving the high combination of analgesics (Group K), see Fig 4A and Table 4. A part of the reason for this happening early post-surgery, is probably the inclusion of local anesthesia (Lidocaine/bupivacaine), since dehiscence occurred regardless if administered alone (J) or in the high (K) or low (L) combination groups. On Day 1 after the surgery, three out of 12 animals had ruptured incision sites in both the low combination (L), carprofen 24 hour (H) and buprenorphine p.o. 72 hour (G) groups, while a total of seven out of 17 of the group receiving the high combination (K) had wound dehiscence, and some several times during the same day. On the following days the numbers declined to only sporadic ruptures (Fig 4A).

**Table 4:** Welfare issues associated with the different treatment groups. Numbers of animals experiencing various welfare problems, with "2/30" meaning that 2 out of a total of 30 animals were affected. Groups and treatment dosages are presented in Table 1. Wound dehiscence; incision wounds at the operation site. Self-mutilation-like behaviour; minor wounds, swelling or erosions on the paw affected by the neuropathic injury. Clinically affected; presence of clinical signs like depression, weight loss exceeding 5%, piloerection, crouched stance, ataxia, decreased food intake or less active appearance.

Group	Wound dehiscence	Self-mutilation-like Behavior	Clinically affected
C–Vehicle, s.c.	2/30 (1 repeated)	2/30	1/30
D–Bup., s.c., 8h	7/30	1/30	-
E–Bup., s.c., 72h	3/12	4/12	-
F–Bup., p.o., 8h	1/12	2/12	-
G–Bup., p.o., 72h	3/13 (1 repeated)	1/13	2/12
H–Carprofen, s.c., 24h	3/12 (1 repeated)	-	-
I–Carprofen, s.c., 72h	2/12	-	2/12
J–Lido/bupi, local, 2-3h	2/12 (1 repeated)	2/12	-
K–High combination	8/17 (4 repeated)	4/17	9/17
L–Low combination	3/12 (3 repeated)	3/12	-

When looking at the tendency to self-mutilation (including minor wounds, swelling or erosions on the paw affected by the neuropathic injury), the group receiving buprenorphine for 72 hours (E) stood out on Day 1 and 2 (Fig 4B), mainly due to three animals showing major swelling of the entire affected hind paw. In general, the self-mutilating behavior occurred on the first two days following surgery, as the animals were adapting to the new circumstances. Effects were also seen in individual animals in both combined treatment groups (K and L) buprenorphine p.o. 8 hours (F), and to a lesser extent in animals receiving local irrigation (J), buprenorphine s.c. 72 hours (G) and vehicle-treated animals (C). The only group of rats not having any problems with self-mutilation-like behaviors at any point in the experiment, were animals in the two carprofen groups (H & I), see Fig 4B. Autotomy (animals biting their toes or the paw off) [30] was not observed at any time in any group.

Another issue was animals showing clinical signs of discomfort. Clinical signs could be weight loss of 5–10% or above, piloerection, porphyria, crouched stance, ataxia, decreased food intake, or inactive appearance (Table 2). Fig 4C clearly shows that a number of animals receiving the high combination analgesic regimen (Group K) experienced clinical signs and discomfort following surgery and the analgesic treatment. Accordingly, the additional cohort of animals treated with a lower dose of buprenorphine combined with carprofen and lidocaine/bupivacaine administered in the same doses as previously (L) exhibited less clinical signs. To a minor extent, some discomfort was also seen in animals receiving buprenorphine p.o. 72 hour (G) with one out of 13 and two out of 13 animals affected on Day 1 and Day 2 post surgery respectively. In addition, the carprofen 72 hour group (I) demonstrated some clinical signs on Day 3–7, observed by individual subjects being slightly pale, losing weight, having a crouched stance and/or piloerection, albeit much less than when compared to the high combination group (K) (Fig 4C).

Five of 17 animals from the group receiving the high combination (K) and one out of 12 from the carprofen 72 hour group (I) reached the pre-defined humane endpoints, and were euthanized and exposed to necropsy. Excessive abdominal peritonitis and ascites were observed in all of them. Abdominal fluid from some affected animals in the high combination group was examined and contained a mixed bacterial flora, indicating that the peritonitis originated from the intestinal tract. A single animal from the high combination group (K) showed clinical signs on Day 3, and improved after this, but fibrous abdominal adhesions indicating earlier peritonitis, was demonstrated at the control-necropsy on Day 32 at the end of the trial. The group receiving the combined treatment with a lower dose of buprenorphine (L) did not show such side effects.

### Fecal immunoreactive corticosterone metabolites (FCM)

The analysis of FCM did not give significant differences for any groups compared to the minimally handled control-group (A), with the exception of buprenorphine 72 hours (E) on Day 3, being significantly (*P<0.05) elevated (data presented in S1 Dataset). When comparing treatment groups with the vehicle-treated animals (C) no significant differences were recorded. There was a tendency of relatively low levels prior to surgery, and an increase in fecal corticosterone-levels peaking at Day 2 following surgery, again followed by a decrease, although this was not statistically significant. Due to inconclusive results, the data is not presented further here, but is provided in S1 Dataset.

## Discussion

A common strategy for reducing incidence, duration and intensity of chronic post-surgery pain, is using preventative analgesia [31]. This concept is based upon the hypothesis, that by reducing afferent input or inhibiting signal transmission at nociceptive synapses, analgesics may prevent development of activity-dependent pain mechanisms like central sensitization and thereby have sustained effects beyond the immediate analgesic effect [32]. This hypothesis has also resulted in many researchers refraining from providing post-operative analgesia when modeling neuropathic pain in their laboratory animal model, in fear of it modifying model pathology. The current data challenge this concept along with both supporting and extending previous findings in this area [13, 14, 16, 33–35]. Pre-emptive and post-operative analgesia utilizing distinct pharmacological mechanisms (NSAIDs, opioid and local-anesthetics) were administered prior to surgery, with coverage for up to 72 hours post nerve-injury, without affecting subsequent emergence of neuropathic pain. Moreover, specific treatments expected to facilitate post-operative recovery actually worsened welfare scores. Accordingly, the pursuit of optimal analgesia in the post-operative care setting may require empirical testing as even the best of intentions of the investigator can apparently have negative consequences for the subjects.

### Do specific analgesic regimens specifically alter the development of mechanical hypersensitivity after SNI?

Although Decosterd & Woolf, 2000 [8] originally reported that all rats subjected to SNI developed mechanical hypersensitivity, other studies have reported the incidence of ‘non-responders’ [14, 36]. The possibility that inclusion of non-responders occurred in our study needs to be carefully considered as it could confound the interpretation of outcome; e.g. does a lack of hypersensitivity reflect that an animal responds to post-operative analgesia or simply a procedural error? In the current study, all groups appeared to develop mechanical hypersensitivity when compared to their baseline and the non-operated control group, albeit with a slower onset to peak of hypersensitivity than previously described [8, 36]. Nevertheless, other groups have also reported a peak in mechanical sensitivity at 14–17 days post SNI [14], with the possibility that incidental damage to the spared sural nerve is a potential confound in this regard [18].

To our knowledge, only a handful of studies have systematically evaluated the utility of systemic post-operative analgesics in preclinical models of neuropathic pain. Comparing oxymorphone, buprenorphine, carprofen and topical EMLA-cream in Spinal Nerve Ligated (SNL) rats Simkins et al. 1998 [13] found that while the developmental time-course for mechanical allodynia differed between treatment groups, none of the analgesics prevented the eventual development of neuropathic pain-like behaviors. Similarly, post-operative administration of buprenorphine, flunixin and fentanyl at different doses and duration for up to 72 hours post nerve injury in SNI rats, had no suppressive effect on the eventual development of neuropathic pain [14]. However, by providing analgesic treatment only once every 24 hours in the 72 hour treatment-period, the possibility that gaps in analgesic coverage (and by association the potential to prevent development of neuropathic pain) cannot be discounted. To address and expand upon this possibility, we (i) provided more extended analgesic coverage as indicated in Table 1 and (ii) combined analgesics with distinct mechanisms which would be expected to interfere with peripheral and/or central pathophysiological pain mechanisms via targeting of mu-opioid receptors, COX enzymes and sodium channel isoforms. Despite these additional considerations, we observed that all SNI rats still developed clear behavioral signs of mechanical allodynia similarly to vehicle-treated animals, and believe this argues strongly for the provision of post-operative analgesia in these circumstances as it apparently does not overtly affect the behavioral outcome.

On balance, it is possible that the underlying neurobiological mechanisms induced by nerve injury might have been subtly affected, which in turn might have consequences for subsequent pharmacological testing in the model. In this latter regard, while there is ample evidence supporting the analgesic efficacy of mu-opioid agonists such as morphine and buprenorphine in animal models of neuropathic pain *per se* [37–39], the specific dose administered could have profound implications for the pharmacological predictivity of a given model. Notably, Curtin et al. 2009 [40] found that whereas 0.05 mg/kg of buprenorphine blocks allodynia for 24 hours in a model of post-incisional pain, a higher dose of 0.1 mg/kg actually produces distal allodynia, likely due to rebound pain mediated by central sensitization. Furthermore, the high dose of buprenorphine produced a long-lasting opioid tolerance indicating that neural adaptations within opioid-sensitive pain circuits are exquisitely sensitive to such small changes in dosing in the post-operative setting [40].

In contrast, NSAIDs are generally accepted to possess minimal efficacy in neuropathic pain [41, 42]. Correspondingly, models such as the SNI-model which involve minimal peripherally localised post injury inflammation in contrast to for example the CCI model appear to be completey insensitive to treatment with a COX-2 inhibitor, even when administered for up to five days post injury using a dose that is anti-hyperalgesic in rats injected with Complete Freund’s Adjuvant in the hindpaw [35]. Accordingly, we have no reason to believe that the post-operative treatment with carprofen in the present study should have any implications for model outcome, besides relieving any discomfort associated with the surgical procedure itself.

A good rationale exists for using local anesthetics (LA) to obtain pre-emptive analgesia prior to injury of nociceptive primary afferents [43, 44]. While application of drugs such as either bupivacaine or lidocaine provides local conduction block of sensory information after SNI or SNL, neuropathic hypersensitivity appears to evolve nonetheless [16, 34]. Other studies challenge these findings, either reporting that lidocaine reduces the development of neuropathic hypersensitivity induced by CCI but not PNL [15] or that local anesthetic block can in fact completely prevent the development of neuropathic hypersensitivity induced by CCI or SNI [17]. It is uncertain why these results differ, although differences in dose/duration and delivery method of anesthetic application have been proposed [43]. Given that we only applied LA to have an effect for a short duration (2–3 hours), either alone or in combination with other analgesics in order to minimize the acute post-operative discomfort associated with the incision-wound, it is probably not surprising that it did not prevent the overall development of neuropathic pain-like behaviors.

A wide range of neuropathic pain models have been developed [5–8, 45], and it is of course possible that some models, depending on mechanisms involved, may be more sensitive to provision of post-operative analgesia. The SNI model was chosen for the current study, as it produces robust and long-lasting neuropathic hypersensitivity localized to a specific sensory area on the affected paw, with a minimal number of non-responders in our facility [8].

In this study we focused on common analgesics to minimize post-operative pain from the surgical site, and did not find that these drugs affected the behavioral outcome of the model. These findings are supported by findings from Yang et al. 2014 [33] who investigated effects of pregabalin, a first line analgesic against neuropathic pain, over 28 days following SNI. Although pregabalin infusion prevented the development of both mechanical and thermal hypersensitivity, once the infusion ended, SNI pregabalin rats exhibited hypersensitivity at a similarly high level as SNI rats receiving vehicle treatment [33]. This clearly illustrates how even first line analgesics against neuropathic pain, cannot prevent its occurrence indefinitely, and advocates for provision of analgesics as a standard post-operative care in animal models of neuropathic pain. However, the implications for human clinical care are less positive, as even aggressive peri-operative analgesic treatment will probably not prevent the emergence of neuropathic pain which may follow many surgeries. This has recently been supported in a meta-analysis performed by Martinez et al. 2017 [46], exploring the effect of peri-operative pregabalin on chronic post-operative pain.

### Do specific analgesic regimens specifically alter body weight, food consumption or welfare endpoints after SNI?

Food and water consumption as well as bodyweight are commonly decreased after surgical manipulation [47], and changes in these parameters are often monitored for evaluation of post-operative wellbeing of rodents. Our expectation was that an appropriate analgesic coverage would minimize the negative effects of surgery on these parameters. However, opioids are known to diminish food consumption [10] presumably due to induction of nausea or sedative actions. This likely explains why all groups administered buprenorphine for 72 hours, irrespective of route or combination with other analgesics (Groups E, G, K and L) displayed various reductions in body weight and food consumption in the immediate post-operative period. Notably, the provision of buprenorphine in Nutella® appeared to induce a slightly prolonged decrease in food intake, also prior to surgery during habituation. We are not sure how much of this effect is due to the extra nutritional energy supplied in the Nutella®, and how much is due to a possible prolonged action of buprenorphine when delivered by this route. The groups given a single administration of carprofen (H) or local application of lidocaine/bupivacaine were the only groups that did not experience a surgery-induced depression of food-intake as observed in all other groups (compared with un-operated groups), indicative of a beneficial effect on post-operative pain. Overall, a slight decrease in food intake compared to body weight occurred in all groups during the experiment, corresponding well with previous findings, that the amount of voluntary ingested food remains quite constant between 8 to 18 weeks of age [48]. Regardless of a depressing effect of opioids on food intake, all groups receiving injections of buprenorphine for 72 hours alone or in combination (group E, K and L), exhibited significantly higher body weight than the vehicle-treated group on the first day after surgery, suggesting some beneficial effects in the immediate post-operative period.

It is likely that the development of complications in SNI rats receiving the high dose of buprenorphine (0.1 mg/kg) combined with carprofen (5.0 mg/kg) and lidocaine/bupivacaine (Group K) is related to some of the mentioned side effects. Post-operative ileus is a common finding in both veterinary and human medicine, and opioids are known to inhibit gastrointestinal propulsion and to prolong post-operative ileus in rats and humans [49]. Common adverse effects of NSAIDs are gastrointestinal-related side effects normally involving ulceration and erosion, and loss of blood or plasma into the peritoneal cavity [50, 51]. These NSAID-related adverse effects do not normally occur following only 72 hours of treatment at the commonly recommended doses, but did unexpectedly occur in one SNI rat receiving only carprofen for 72 hours, and with a higher prevalence when combined with the high dose of buprenorphine. The latter finding presumably reflects diminished buprenorphine-mediated gut motility impacting upon the carprofen-mediated reduction of gastrointestinal-protective properties of cyclooxygenases, leading to leakage of microorganisms giving infection and increased peritoneal fluid.

Of the various regimens tested other welfare-related adverse side effects were observed. However, autotomy, a behaviour where animals start to bite their toes off [30] was not observed in the current study *per se*. We did though note that the local application of lidocaine/bupivacaine increased the prevalence of wound dehiscence in the immediate post-operative recovery period, presumably due to an urge to investigate the fresh incision area which lacked a nociceptive signaling response as a consequence of the treatment [17]. Animals in the combined treatment groups receiving carprofen and buprenorphine in addition to the lidocaine/bupivacaine, continued this behavior throughout the treatment period. This emphasizes the need to balance the engagement of analgesic and anesthetic mechanisms post-operatively, as the increased sensitivity around the incision site has some beneficial effects by encouraging the animal to refrain from manipulating the fresh incision wound.

### Do specific analgesic regimens affect surgery-induced stress levels after SNI?

Measurement of excreted Fecal Corticosterone Metabolites (FCM) has been shown to be an accessible and reliable method for assessing effects of various stressors on glucocorticoid levels in several species including rats [52, 53]. Moreover, administering buprenorphine through voluntary ingestion in a sticky nut-paste has been shown to reduce a post-operative-induced increase in plasma corticosterone levels [54]. In light of these observations, we expected to see differences in FCM between the different treatment regimens and the von Frey tested control group (B) versus the minimally handled control group (A), enabling us to extrapolate to (i) a preferred analgesic regimen from a welfare perspective and (ii) to what degree handling and testing accounted for the overall FCM-response in the operated groups. To overcome the diurnal fluctuations in regards to excretion of FCM, and keeping in mind the significant variation between concentration of markers in individual fecal pellets or samples [55, 56], a sampling interval of 24 hours duration was implemented. This gives an overall picture of the stress level in the different cages from day to day, but may not detect small occasional changes in FCM [53, 57].

Our data unfortunately failed to reveal any significant differences between the different treatment or control groups. We cannot provide a definitive reason for this, but did note that there were a number of sporadic outliers in the various groups, which we could not easily explain outside of analytical error or possible fecal metabolite decomposition, and therefore were not comfortable excluding from the overall analysis. Alternatively, the repeated handling of the SNI rats for purposes of measuring von Frey thresholds and changing bedding might have acted as an intermittent stressor under the conditions tested. Of course, this being the case it would then have been expected to see an overall effect of treatment versus minimally handled rats, but with the rats pair-housed throughout the study, we obtained a relatively low treatment sample size, precluding such a comparison based on simple lack of power. Nevertheless, there was a general tendency for FCM to be lower prior to surgery, before increasing in the immediate post-operative period and then declining towards baseline levels at the end of the first week. This temporal profile correlated reasonably well with our expectations.

## Conclusions

In summary, the present study demonstrates that the selected analgesic treatment regimens did not prevent the development of neuropathic hypersensitivity after SNI. However, the study also highlights that associated behavioral sequelae reflecting negative facets of animal welfare, such as dehiscence of incision wounds, were clearly affected by some of the regimens. Correspondingly, some of the associated treatments such as acute carprofen or local application of lidocaine/bupivacaine seemed to have positive effects on food intake immediately post-surgery, and repeated injections of buprenorphine (alone or in combination) lead to a significant higher body weight compared with animals given vehicle treatment. Moreover, we found that a combination of a relatively high dose of buprenorphine and carprofen administered over 72 hours in parallel with a local analgesic irrigation of the injury site caused significant adverse effects with excessive peritonitis. Accordingly, we would suggest that post-operative analgesia needs to be balanced with welfare issues of the test subject. Whilst further studies are needed to elucidate if post-operative analgesia produces neurobiological changes that may affect the integrity of the model, or if post-operative analgesia would have disease-modifying effects in other models of neuropathic pain, we see no reason for not providing at least a minimal level of post-operative analgesia in similar animal models of peripheral nerve injury.

## Supporting information

S1 Dataset All data obtained during this study. (XLSX)
